# Nailed it? A diagnostic puzzle in an infant

**DOI:** 10.1016/j.jdcr.2026.02.016

**Published:** 2026-02-13

**Authors:** Rita El Jbeily, Heather Gochnauer, Maria Gnarra Buethe, Lawrence F. Eichenfield, David Schairer

**Affiliations:** a Michigan State University College of Human Medicine, East Lansing, Michigan; b Division of Pediatric and Adolescent Dermatology, Rady Children's Hospital San Diego, San Diego, California; c Department of Dermatology, University of California San Diego School of Medicine, La Jolla, California; d Division of Dermatology, Children’s Hospital of Orange County (CHOC), Orange, California; e Department of Dermatology, University of California Irvine, Irvine, California; f Department of Pediatrics, University of California San Diego School of Medicine, La Jolla, California

**Keywords:** blistering disorders, general dermatology, genetics, junctional epidermolysis bullosa, medical dermatology, nails, pediatrics

## Case description

**Fig 1:**
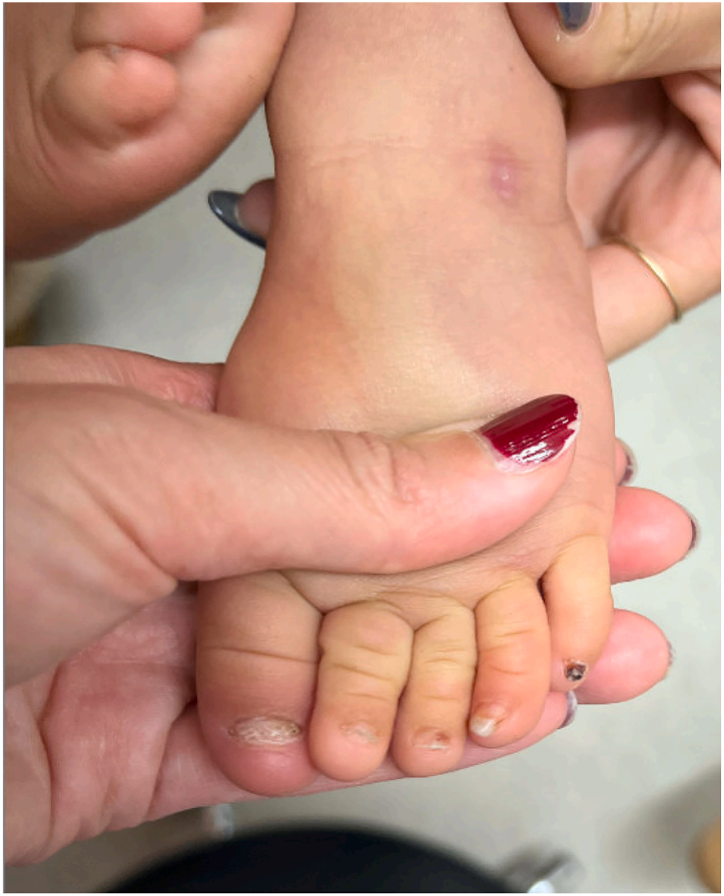
Clinical findings of toenail dystrophy and subungual hemorrhage at 14 months old.

**Fig 2:**
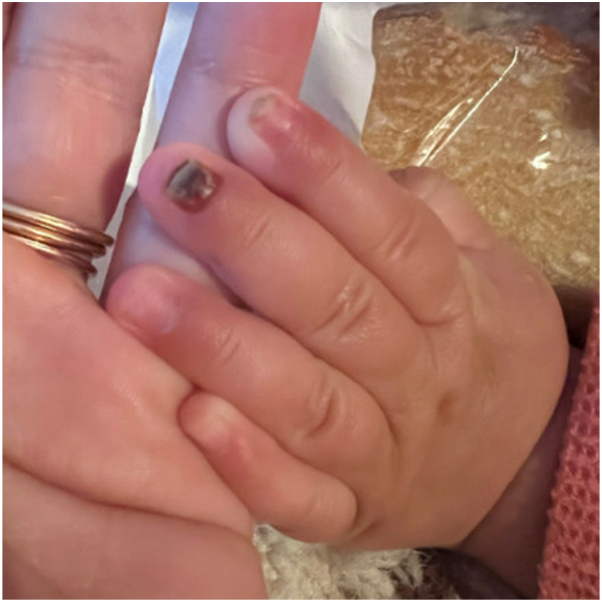
Patient's presentation to pediatrician at 7 months old for nail problems with fingernail dystrophy and subungual hemorrhage.

An 8-month-old otherwise healthy female presented to dermatology clinic with a 2-month history of fingernail and toenail dystrophy. Nail changes were persistent despite a 10-day course of cephalexin prescribed by the patient’s pediatrician. Physical examination revealed subungual hyperkeratosis, onycholysis, and proximal paronychia affecting nearly all toenails and fingernails (Figs 1 and 2). The remainder of the exam was unremarkable. A nail clipping was obtained for Periodic acid-Schiff staining, which revealed debris with neutrophils and gram positive cocci. A fungal culture was negative. Genetic testing was completed.

**Fig 3:**
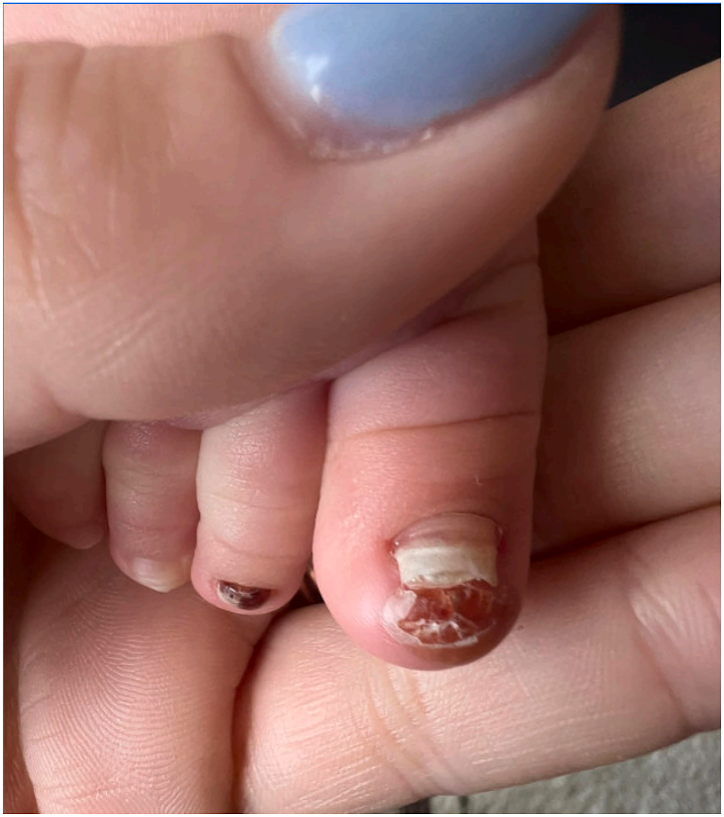
Patient's presentation to pediatrician at 7 months old for toenail dystrophy and localized blisters on the tips of toes.

**Fig 4:**
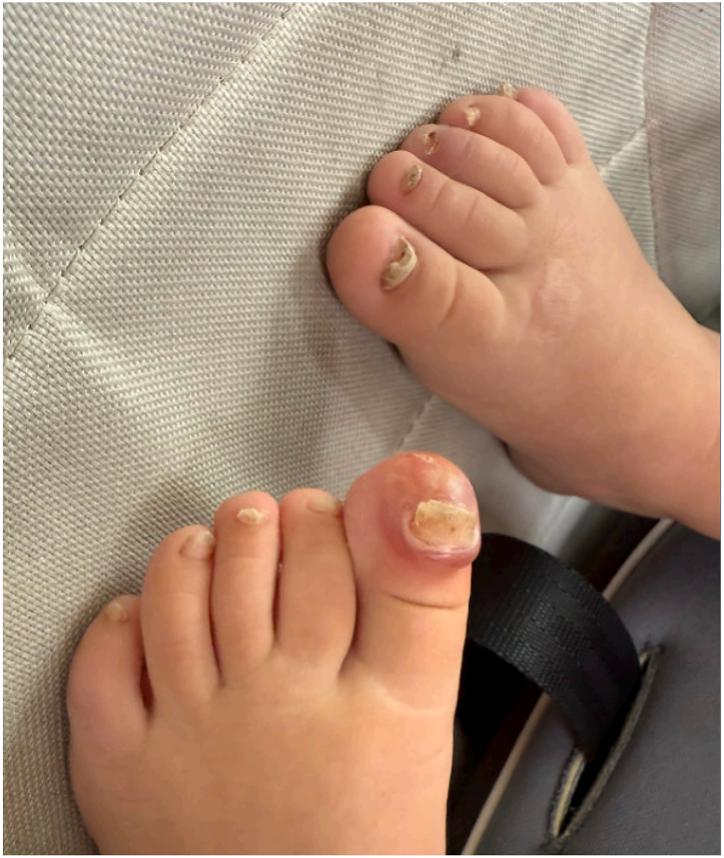
Bilateral toenail dystrophy with localized blisters present on the tips of toes and pads of great toes noted at 18 months old.

The patient returned to clinic 5 weeks later with no improvement and new, painless blisters on the tips of her toes despite a second 10-day course of cephalexin treatment (50 mg/kg divided three times daily) and mupirocin ointment. On follow-up at 18 months of age, the patient had additional blisters on the pads of her great toes; however, the patient’s nails had shed and regrown without complications. There was no family history of similar lesions (Figs 3 and 4).

### Question: What is the most likely diagnosis based on the patient’s presentation?

**A.** Bullous impetigo
**B.** Pemphigoid vulgaris
**C.** Chronic bullous disease of childhood (linear IgA bullous dermatosis)
**D.** Severe junctional epidermolysis bullosa (JEB)
**E.** Localized JEB

## Discussion

**E.** A diagnosis of localized JEB was made based on physical examination findings and genetic testing.

Major forms of JEB include severe (formerly Herlitz), intermediate (formerly non-Herlitz), and localized. Due to overlapping and variable clinical findings among EB subtypes, genetic testing is essential for accurate diagnosis. Severe JEB classically presents as granulation tissue around the oral and nasal cavities, and on the distal fingers and toes. Patients may have mucosal involvement of the mouth, upper respiratory system, bladder, urethra, and corneas.^1^ However, milder forms of JEB can present with only nail involvement and limited skin blistering.^2^

Genetic testing in our patient revealed compound COL17A1 heterozygous mutations, consistent with several other studies which have reported mutations in the COL17A1 gene associated with mild, localized forms of JEB.^3^^,^^4^ JEB is often associated with the most severe subtype, and it is important to remember that mild and localized forms exist. This patient’s presentation of JEB was limited almost exclusively to nail findings without widespread involvement. The patient did not have any dental concerns or issues with oral intake. Otolaryngology evaluated the patient for dysphonia and intermittent voice hoarseness and concluded it was likely attributable to JEB. No mucosal bullae or skin sloughing was seen on flexible laryngoscopy and the patient’s voice improved. Localized JEB may be underdiagnosed or misclassified as EB simplex based on clinical findings alone; mild mucosal involvement can be an important clue to the actual EB subtype.

Management of symptoms is focused on preventing trauma due to skin fragility which can be accomplished by patient and caregiver education on handling.^1^ Treatment involves draining and dressing blisters, antibiotics, and antiseptics as needed.^1^ Published reports describe adults with localized JEB, indicating this mild phenotype is compatible with survival into adulthood.^3^^,^^4^ Localized JEB should be included as a differential diagnosis when evaluating patients with isolated nail dystrophy and/or blisters.

## Conflicts of interest

None disclosed.
